# Abdominal Aortic Thrombosis in a Preterm Baby With Congenital Diaphragmatic Hernia

**DOI:** 10.7759/cureus.89401

**Published:** 2025-08-05

**Authors:** Vivek Goyal, Neha Jain, Monica Goyal

**Affiliations:** 1 Pediatrics and Neonatology, Janki Children Hospital, Hisar, IND; 2 Anesthesiology, Om Prakash (OP) Jindal Institute of Medical Sciences, Hisar, IND

**Keywords:** anticoagulation, congenital diaphragmatic hernia (cdh), pphn, thromboembolic events, umbilical arterial catheter

## Abstract

Congenital diaphragmatic hernia (CDH) is a serious congenital anomaly often associated with pulmonary hypoplasia and persistent pulmonary hypertension of the newborn (PPHN). Central vascular access such as umbilical arterial catheters (UACs) is routinely used in neonatal intensive care but is associated with the risk of vascular complications, including thromboembolic events.

We present a case of preterm dichorionic diamniotic (DCDA) twins born at 34 weeks of gestation with antenatally diagnosed CDH. The baby developed severe respiratory distress requiring high-frequency oscillatory ventilation (HFOV), inhaled nitric oxide (iNO), and sildenafil infusion. Hemodynamic instability required inotropic support and UAC placement. Surgical repair of CDH was performed on the fifth day of life. Postoperatively, the baby developed iliofemoral and renal artery thrombosis, leading to global infarction of the left kidney.

Anticoagulation was initiated with heparin and transitioned to enoxaparin. The neonate showed significant clinical improvement and was discharged in stable condition with preserved right renal function.

## Introduction

Congenital diaphragmatic hernia (CDH) is a rare but life-threatening anomaly. The overall prevalence of CDH is 2.3 per 10,000 births [1]. This condition leads to varying degrees of pulmonary hypoplasia and pulmonary hypertension, significantly impacting neonatal morbidity and mortality [2]. Umbilical arterial catheter (UAC) placement, though frequently used in the neonatal intensive care unit for monitoring and blood sampling, carries risks of vascular complications, including thromboembolism. Symptomatic aortic thrombosis in neonates is a rare condition, with an estimated incidence ranging from 0.1 to 1.1 per 100,000 live births [3,4]. Neonates are predisposed to a prothrombotic state, and several factors can precipitate arterial thrombosis. These include endothelial injury, sluggish blood flow, coagulation factor imbalances, and the use of central vascular catheters. Among these, the presence of an indwelling vascular catheter remains the most significant risk factor, contributing to nearly 90% of all arterial thrombotic events in this population [5]. UAC is associated with clinically significant vascular obstruction requiring intervention in approximately 1% of cases [6].

Arterial thrombosis resulting in renal infarction is an extremely rare complication in neonates with CDH, with very limited reports in the literature. We present a rare case of a preterm twin with antenatally diagnosed CDH who developed severe persistent pulmonary hypertension of the newborn (PPHN) and hemodynamic instability, underwent surgical repair, and subsequently developed iliofemoral and renal artery thrombosis, leading to unilateral renal infarction. This case highlights the importance of vigilance for vascular complications in neonates with multiple invasive interventions and the challenges in balancing anticoagulation therapy in a post-surgical setting.

## Case presentation

Dichorionic diamniotic (DCDA) twins were delivered at 34 weeks of gestation via lower segment cesarean section (LSCS) to a 31-year-old primigravida mother. The antenatal course was notable for pregnancy-induced hypertension (PIH), managed with labetalol. Twin 1 was antenatally diagnosed with CDH. The baby was born with a birth weight of 2.1 kg and had an immediate cry. However, the baby soon developed severe respiratory distress and was referred to our center at four hours of life (HOL) on bag-and-tube ventilation.

On examination, the baby was hypoxic with SpO₂ of 60% on bag-and-tube ventilation, respiratory rate (RR) of 54/min, and heart rate (HR) of 146/min. Auscultation revealed markedly reduced air entry on the left side. The abdomen was distended but soft on palpation.

**Figure 1:**
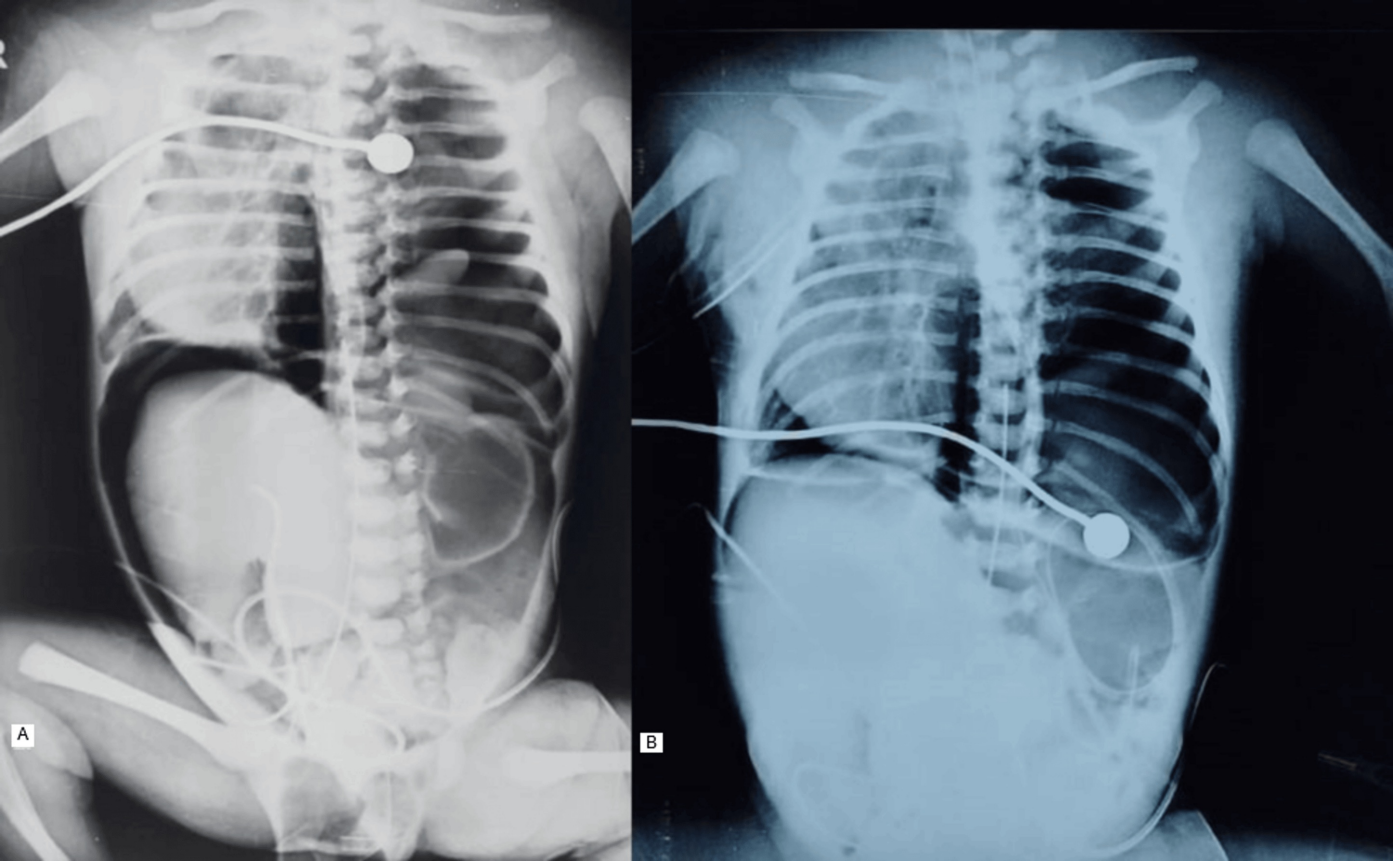
(A, B) Chest and abdominal radiographs (A) Chest and abdominal radiograph (at 6 HOL) showing left-sided congenital diaphragmatic hernia with associated dextrocardia, right-sided pneumothorax, pneumomediastinum, and pneumopericardium. (B) Follow-up radiograph (at 9 HOL) after insertion of an intercostal drainage tube into the right pleural space showing resolution of the pneumoperitoneum.

The baby was placed on mechanical ventilation (synchronized intermittent mandatory ventilation (SIMV) mode). Chest and abdominal radiographs at six HOL showed findings of left-sided CDH, associated dextrocardia, right-sided pneumothorax, pneumomediastinum, and pneumoperitoneum (Figure 1A). An intercostal drainage (ICD) tube was placed in the right pleural space, following which resolution of pneumoperitoneum was seen at nine HOL (Figure 1B).

**Figure 2:**
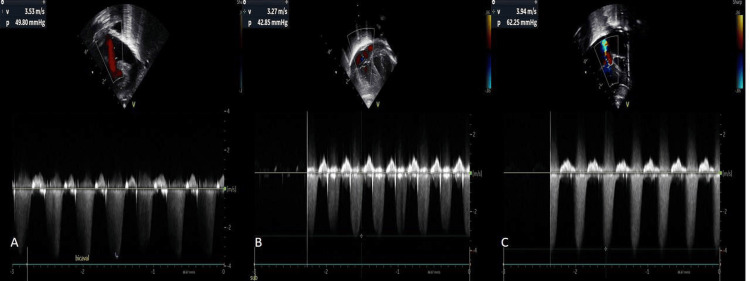
(A-C) Serial echocardiographic images showing tricuspid regurgitation (TR) jet velocities and gradients (A) Bicaval view showing TR jet with a peak gradient of 49.80 mmHg. (B) Subcostal view showing TR jet with a reduced gradient of 42.85 mmHg. (C) Re-elevation of the TR jet gradient to 62.25 mmHg.

The baby had persistent respiratory distress and frequent desaturation episodes. Echocardiography performed at seven HOL revealed features of PPHN, with a tricuspid regurgitation (TR) jet of 49.80 mmHg and a right ventricular systolic pressure (RVSP) of 54.80 mmHg (Figure 2A). In view of worsening oxygenation, the baby was shifted to high-frequency oscillatory ventilation (HFOV), and sildenafil infusion was initiated.

At 17 HOL, the baby developed signs of poor perfusion, including feeble peripheral pulses and prolonged capillary refill time. Inotropic support was initiated with dobutamine at 10 µg/kg/min and noradrenaline at 0.1 µg/kg/min. A UAC was placed for continuous invasive blood pressure monitoring. A prophylactic infusion of unfractionated heparin was started at 0.5 units/kg/hour to maintain catheter patency and reduce the risk of thrombotic complications.

Gradually, the respiratory status improved, so the baby was shifted to conventional ventilation. Pulmonary pressures started decreasing with the lowest TR jet being 42.85 mmHg; hence, sildenafil infusion was continued (Figure 2B). Inotropes were tapered based on clinical improvement and discontinued by the fourth day of life (DOL).

Surgical repair of the CDH was performed on DOL-5. Postoperatively, the baby maintained adequate oxygen saturation on conventional ventilation. However, the baby developed hemodynamic instability, evidenced by feeble peripheral pulses and a prolonged capillary refill time. Inotropic support was restarted to maintain adequate perfusion.

On DOL-8, the baby developed worsening respiratory distress and was transitioned back to HFOV. Repeat echocardiography revealed elevated pulmonary pressures with a TR jet of 62.25 mmHg (Figure 2C).

Inhaled nitric oxide (iNO) therapy was initiated at 20 parts per million (ppm), leading to significant clinical improvement. iNO was gradually weaned and discontinued over the next 4-5 days.

During the hospital stay, the baby had a positive septic screen and was managed with intravenous antibiotics. Cerebrospinal fluid analysis was normal, and meningitis was ruled out. Table 1 shows the laboratory investigations.

**Table 1:** Laboratory investigations CRP: C-reactive protein; PT: prothrombin time; INR: international normalized ratio; APTT: activated partial thromboplastin time; DOL: day of life

Parameters	DOL-2	DOL-3	DOL-6	DOL-8	DOL-9	DOL-11	DOL-13	DOL-16	DOL-18	DOL-20	DOL-23	Reference values
Hemoglobin	18.7	17.8	8.6	11.7	12.2	10.5	10.8	12.0	10.6	8.9	12.9	12-16 g/dL
Total leukocyte count	10.8	12.3	9.6	10.0	11.1	25.3	21.3	27.6	22.6	19.2	25.1	4-12 × 10^9^/L
Platelet count	217	133	83	108	77	72	72	84	118	141	166	150-450 × 10^9^/L
CRP	NA/-	5.1	7.1	108.4	88.8	113.6	76.4	38.6	81.7	23.3	4.1	<6 mg/dL
Urea	NA/-	23	NA/-	29	NA/-	37	NA/-	21	NA/-	NA/-	NA/-	16-43 mg/dL
Creatinine	NA/-	NA/-	NA/-	NA/-	NA/-	0.80	NA/-	0.70	NA/-	NA/-	NA/-	0.70-1.40 mg/dL
Sodium	NA/-	132	132	NA/-	13A1	131	143	NA/-	NA/-	NA/-	136	135-145 mmol/L
Potassium	NA/-	4.2	4.8	NA/-	2.65	4.5	3.2	NA/-	NA/-	NA/-	3.5	3.5-5.5 mmol/L
Ionized calcium	NA/-	4.7	4.9	NA/-	4.4	4.4	4.6	NA/-	NA/-	NA/-	4.6	4.5-4.9 mg/dL
PT/INR	NA/-	NA/-	17.1/1.36	NA/-	NA/-	20.5/1.63	24.7/1.96	19.5/1.55	NA/-	NA/-	NA/-	12-21/1.0-1.5
APTT	NA/-	NA/-	98.0	NA/-	NA/-	48.9	>120	50.3	NA/-	NA/-	NA/-	70-145

**Figure 3:**
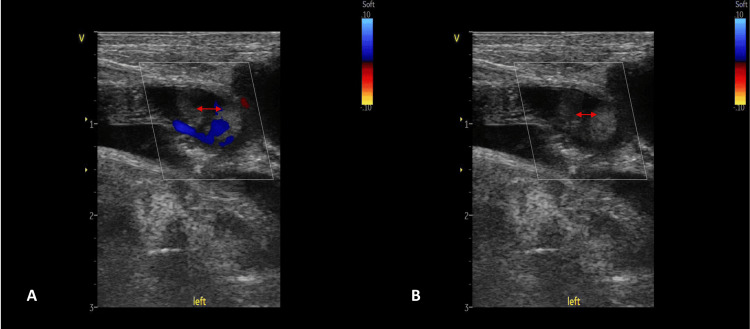
(A, B) Doppler ultrasound of the left femoral artery showing thrombus formation (A) Color Doppler ultrasound image of the left femoral artery showing an intraluminal thrombus (red double-headed arrow) with partial color flow (blue and red signals) surrounding the thrombus. (B) Ultrasound image of the same artery segment. The thrombus (red double-headed arrow) appears as a hypoechoic structure within the vessel lumen.

On DOL-11, the baby developed absent pulses and undetectable blood pressure in the left lower limb. The limb appeared discolored and was cool to the touch. An urgent Doppler ultrasound study revealed thrombotic occlusion at the bifurcation of the left iliofemoral artery. Figure 3A shows the color Doppler ultrasound image of the left femoral artery demonstrating an intraluminal thrombus with partial color flow surrounding the thrombus. Figure 3B reveals the hypoechoic structure within the vessel lumen, suggestive of a thrombus.

Video 1 shows real-time Doppler ultrasound of the left femoral artery.

**Video 1:** Real-time Doppler ultrasound of the left femoral artery Doppler ultrasound of the left femoral artery showing intraluminal thrombus with disrupted color flow pattern. Note the absence of color signal over the thrombus region.

**Figure 4:**
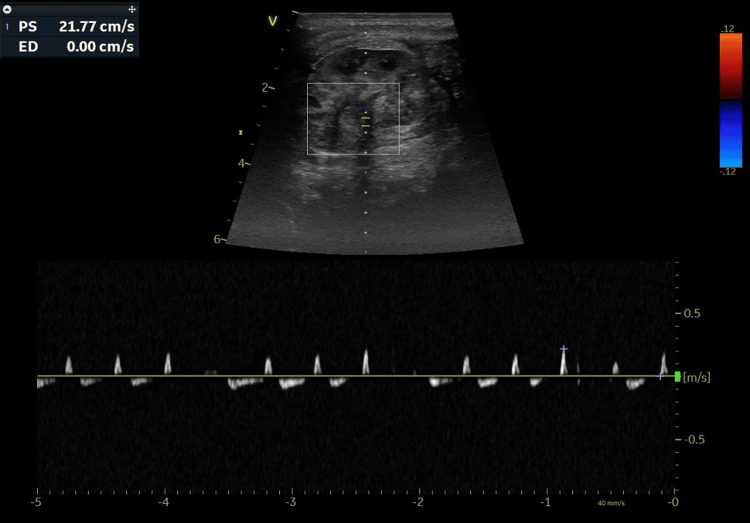
Doppler ultrasound of the left renal artery Spectral Doppler waveform of the left renal artery showing significantly reduced peak systolic velocity (PSV = 21.77 cm/s) with reversed end-diastolic velocity.

Additionally, significantly reduced renal perfusion was observed on the color Doppler ultrasound of the left kidney (Video 2) with reduced peak systolic velocity and reversed end-diastolic flow noted in the vessel (Figure 4).

**Video 2:** Real-time color Doppler ultrasound of the left kidney demonstrating reduced renal perfusion The renal parenchyma shows markedly diminished color flow signals, suggestive of impaired arterial blood supply.

The UAC was removed immediately, and therapeutic anticoagulation was initiated with unfractionated heparin infusion at a dose of 28 units/kg/hour. Close monitoring of urine output was done. Treatment with thrombolytic drugs or thrombectomy was not considered in view of recent major surgical intervention.

Clinical improvement was noted by the second to third day of heparin infusion, with the left lower limb becoming warm and pulses gradually becoming palpable. Blood pressure and peripheral perfusion in the affected limb normalized.

On DOL-14, the coagulation profile showed deranged levels; hence, subcutaneous enoxaparin was started in place of heparin infusion. CT angiography performed on DOL-25 revealed partial thrombosis of the infrarenal abdominal aorta along with mild, diffuse narrowing of the iliac vessels. Additionally, left renal arterial thrombosis leading to global infarction of the left kidney was noted. Genetic thrombophilia workup was planned but could not be done due to family reservations.

The baby showed gradual improvement in respiratory status and was shifted back to conventional ventilation on DOL-11. Sildenafil infusion was tapered and discontinued on DOL-13. The baby was successfully extubated from SIMV to continuous positive airway pressure (CPAP) by DOL-16, and CPAP was weaned off by DOL-22. ICD was removed on DOL-13 once complete resolution of pneumothorax was confirmed. Gavage feeding was initiated on DOL-13, which was well tolerated and gradually advanced. Direct breastfeeding was successfully established in the later course.

By DOL-29, the baby was hemodynamically stable with good pulses in all four limbs, normotensive, maintaining saturation on room air, and accepting breastfeeds well. The baby was discharged on subcutaneous enoxaparin with close follow-up, and levels of anti-factor Xa were monitored. The level of anti-factor Xa was 0.12 IU/mL on DOL-28.

Follow-up Doppler ultrasonography showed partial thrombosis of the infrarenal abdominal aorta with abrupt narrowing. The right and left iliac arteries (common, external, and internal) demonstrated biphasic flow with normal peak systolic velocities. Renal function, urine output, blood pressure, and growth parameters remained within normal limits during follow-up visits.

## Discussion

This case underscores the complex interplay between congenital anomalies, hemodynamic instability, and vascular complications in a preterm neonate. CDH is often associated with pulmonary hypoplasia and PPHN, necessitating aggressive respiratory and hemodynamic support. Our patient required multiple modalities, including mechanical ventilation, HFOV, sildenafil infusion, and iNO therapy to manage PPHN.

In our case, the baby initially had right-sided pneumothorax, pneumomediastinum, and pneumoperitoneum. These improved with ICD not requiring any other interventions. Pathophysiology of pneumoperitoneum could possibly be secondary to pneumomediastinum or pneumothorax with extension of free air through CDH [7].

There is no established association between CDH and arterial thrombosis. The incidence of symptomatic arterial thrombosis in neonates ranges from 0.1 to 1.1 per 100,000 live births [3,4]. Clinical manifestations can vary and may include irritability, intestinal malperfusion, renal failure, or acute limb-threatening ischemia. Renal artery thrombosis is a particularly rare but serious complication, with long-term implications including hypertension and impaired renal function.

Arterial thrombosis most commonly occurs secondary to UAC. Other contributing factors include prematurity, sepsis, dehydration, and underlying prothrombotic conditions [8]. In our case, prophylactic heparin infusion was initiated at a rate of 1 unit/hour (equivalent to 0.5 unit/kg/hour of unfractionated heparin) for UAC. Despite this, it proved insufficient in preventing thrombosis.

Preterm and critically ill neonates are more susceptible to catheter-related complications due to lower levels of natural anticoagulants and an increased prothrombotic tendency. The risk of thrombosis increases with the duration of catheterization [9]. Notably, even in the absence of inherited thrombophilic conditions, neonates are inherently at higher risk of thrombotic events.

Early recognition of absent limb pulses and Doppler confirmation allowed prompt removal of the catheter and initiation of anticoagulation therapy. Fortunately, limb ischemia resolved with heparin, and long-term enoxaparin therapy was well tolerated.

The management of aortic thrombosis in neonates remains a subject of debate. Available treatment options include expectant management, therapeutic anticoagulation, thrombolytic therapy, and surgical thrombectomy. Commonly used thrombolytics include urokinase, streptokinase, and recombinant tissue plasminogen activator (r-TPA)-the latter being the only thrombolytic agent recommended for pediatric use. However, data on its use in preterm neonates remain limited.

Surgical thrombectomy is rarely performed in premature infants due to limited experience and is typically reserved for cases with imminent abdominal visceral ischemia, renal failure, or limb-threatening ischemia. In our case, the choice to avoid thrombolytic therapy or thrombectomy was justified due to the recent surgical repair, balancing the risk of hemorrhage against the benefit of intervention. This conservative approach proved effective in achieving clinical stabilization.

Long-term complications of aortic thrombosis may include persistent thrombus, renovascular hypertension, limb length discrepancies, functional impairment, and tissue loss. Mortality rates associated with neonatal aortic thrombosis have been reported between 4% and 18% [10]. In our study, the limitations include the lack of genetic thrombophilia workup and follow-up Doppler images.

## Conclusions

This case highlights the multifaceted management required in neonates with CDH, especially when complicated by persistent pulmonary hypertension and vascular thrombosis. The timely recognition and management of iliofemoral and renal artery thrombosis were crucial in preserving limb viability and renal function. Clinicians should maintain a high index of suspicion for thrombotic complications in neonates with central lines and consider early imaging in cases of limb hypoperfusion. Long-term follow-up is essential to monitor for sequelae related to renal infarction and vascular compromise. This case reinforces the importance of coordinated, multidisciplinary neonatal care in achieving favorable outcomes in high-risk infants and the importance of early Doppler in suspected thrombotic events post-UAC.
